# Extraction of Oil from Wheat Germ by Supercritical CO_2_

**DOI:** 10.3390/molecules14072573

**Published:** 2009-07-15

**Authors:** Alessandra Piras, Antonella Rosa, Danilo Falconieri, Silvia Porcedda, Maria. A. Dessì, Bruno Marongiu

**Affiliations:** 1 Dipartimento di Scienze Chimiche, Università degli Studi di Cagliari, Cittadella Universitaria di Monserrato, SS 554, km 4.500 09042 Cagliari, Italy; E-mails: apiras@unica.it (A.P.); danilo.falconieri@tiscali.it (D.F.); porcedda@unica.it (S.P.); 2 Dipartimento di Biologia Sperimentale, Sezione di Patologia Sperimentale, Università degli Studi di Cagliari, Cittadella Universitaria di Monserrato, SS 554, km 4.500 09042 Cagliari, Italy; E-mails: anrosa@unica.it (A.R.); dessima@unica.it (M-A.D.)

**Keywords:** wheat germ oil, vitamin E, fatty acid, supercritical extraction, carbon dioxide

## Abstract

This study examined the supercritical fluid extraction of wheat germ oil. The effects of pressure (200-300 bar at 40 °C) and extraction time on the oil quality/quantity were studied. A comparison was also made between the relative qualities of material obtained by SFE and by organic solvent extraction. The extracts were analyzed for α-tocopherol and polyunsaturated fatty acid content. The maximum wheat germ oil yield at about 9% was obtained with supercritical carbon dioxide extraction at 300 bar, while fatty acid and α-tocopherol composition of the extracts was not remarkable affected by either pressure or the extraction method.

## 1. Introduction

Fats and oils play an important role in the food industry and are essential part of human nutrition. Vegetable oils provide fat-soluble vitamins such as vitamins A, D, E, and K, and are also source of essential unsaturated fatty acids, which cannot be synthesized by the human body. In order to meet nutritional requirements, new vegetable oil resources are being sought as sources of these vitamins and essential fatty acids.

Wheat germ oil has the highest tocopherol content of all vegetable oils, up to about 2,500 mg/kg, and also the highest content of α-tocopherol, which represents around 60% of the total content [1]. Wheat germ oil is also highly valued for its high content of unsaturated fatty acids: it has about 80%, consisting mostly of linoleic (18:2) and linolenic (18:3) acids [2], both of which are of great importance in human metabolism and cannot be synthesized by the organism. They are precursors of a group of hormones called prostaglandins, which play an important role in muscle contractions and in the proper healing of inflammatory processes [3]. Furthermore, linoleic acid [4] helps to eliminate cholesterol and is a precursor of cell membrane phospholipids [4].

Wheat germ is a by-product of the wheat milling industry. Germ constitutes about 2-3% of the wheat grain and can be separated in a fairly pure form from the grain during the milling process [5]. Wheat germ contains about 11% oil [6]. Wheat germ oil is used in products such as foods, biological insect control agents, pharmaceuticals and cosmetic formulations [7]. Polyunsaturated fatty acids and bioactive compounds are prone to oxidation and degradation under the conditions used for conventional edible oil extraction and refining methods [8]. 

Solvent extraction is a common method of extraction of oils from vegetable matter. In recent years supercritical fluid extraction (SFE) has received increased attention as an important alternative to conventional methods. Supercritical fluids have adjustable extraction characteristics due to their density, which can be controlled by changes in pressure or temperature. In addition, other properties such as low viscosity, high diffusivity and low surface tension enhance the solute mass transfer from inside a solid matrix. Supercritical carbon dioxide (SC-CO_2_), being nontoxic, nonflammable, inexpensive and easily separable from the extracts, has been the most frequently used extractant in the food and pharmaceutical industries [9,10,11]. Furthermore, the low critical temperature of carbon dioxide allows extraction of thermolabile compounds without degradation. Use of supercritical fluid such as carbon dioxide for extraction of wheat germ oil has previously been reported by several research groups [12,13,14,15,16,17,18]. Amoung these, Panfili *et al*. [12] studied the effect of pressure, temperature, extraction time and wheat germ oil particle size on oil extraction yields. They found that fatty acids composition not change while the greatest concentration of α-tocopherol was found in the fraction sample at 75 min (P= 38 MPa, T= 55 °C, particle size= 0.35 mm).

According to Taniguchi *et al*. [13] the α- and β-tocopherol content of supercritical carbon dioxide extracted oil were similar to those of hexane extracted oil. However, Molero Gomez and Martinez de la Ossa [14] reported higher tocopherol content in the SC-CO_2_ extracted material, as compared to that of the hexane extracted oil. Shao *et al*. [15] found that the fatty acid composition of germ oil extracted by SFE was similar to the oil extracted by hexane. Eisenmenger and Dunford [16] also showed that methods used for oil extraction and refining did not have a remarkable effect on the fatty acid composition of the oil, whereas SC-CO_2_ extracted oil had a higher tocopherol content than that of commercially hexane extracted oil. Ge *et al*. [17] reported that the yield of α-tocopherol extracted by SFE-CO_2_ were much higher than those prepared by chloroform/methanol extraction. However, the solution of chloroform/methanol showed a stronger solvent power to extract β-tocopherol form wheat germ. Finally, Zacchi *et al*. [18] found that petroleum ether extracted oil showed the highest values for fatty acids and tocopherol contents. 

The aim of this work is to study the effects of pressure and extraction time on the extraction efficiency and oil quality.

## 2. Results and Discussion

**Figure 1:**
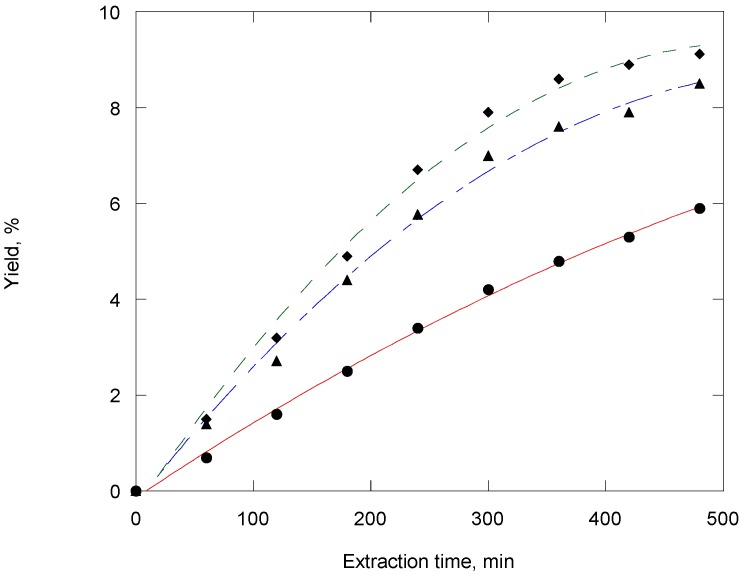
Effect of pressure on the extraction yield of wheat germ oil: ●, 200 bar and 40 °C; ▲, 250 bar and 40 °C; ♦, 300 bar and 40 °C.

**Figure 2:**
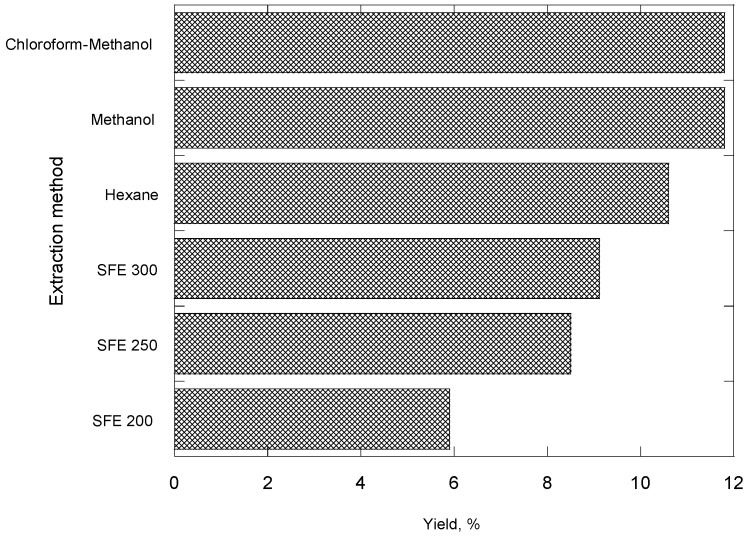
Effect of extraction method on the yield of wheat germ oil.

Figure 1 shows the influence of operating pressure on the extraction yield. An extraction time of 8 h at 300 bar gives wheat germ oil yield above 9% (w/w), oil recovery about 80%. The SC-CO_2_ gives an extraction yield not significantly different from organic solvent extraction. A comparison is made between the relative qualities of the oils produced by SC-CO_2_ and by organic solvent extraction (hexane, methanol, chloroform-methanol 2:1 mixure) in Figure 2. The difference is interpreted that the organic solvents are much less selective than CO_2_ in the extracted oil and produce oil containing some undesirable compounds.

**Figure 3:**
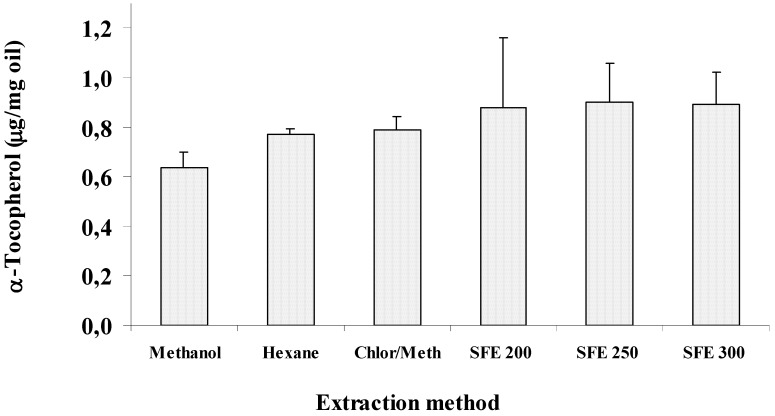
α-Tocopherol amount (expressed as μg/mg oil) measured in wheat germ oil samples obtained by SC-CO_2_ (200, 250, and 300 bar) and organic solvent extraction (hexane, methanol, and chloroform-methanol).

Figure 3 shows the concentration of α-tocopherol (expressed as μg/mg oil) measured in wheat germ oil samples obtained by SC-CO_2_ (200, 250, and 300 bar, temperature of 40 °C, extraction time of 8 h) and organic solvent extraction (hexane, methanol, and chloroform-methanol). There was no considerable variation in α-tocopherol content among oil samples obtained by different extraction procedures, except for oil from MeOH extraction, that exhibited the lowest α-tocopherol amount.

**Figure 4:**
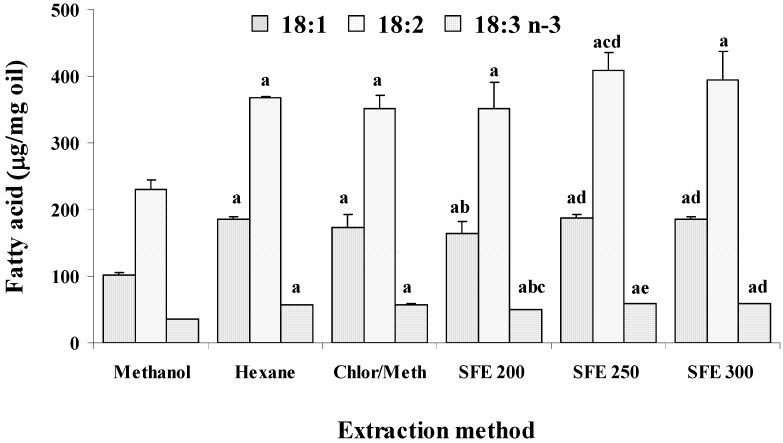
Values (expressed as μg/mg oil) of oleic (18:1), linoleic (18:2), and linolenic (18:3 n-3) acids measured by high-performance liquid chromatography (HPLC-DAD) method in wheat germ oil samples obtained by SC-CO_2_ (200, 250, and 300 bar) and organic solvent extraction (hexane, methanol, and chloroform-methanol). p< 0.05, ^a^ = versus methanol, ^b^hexane, ^c^chloroform-methanol, ^d^200 bar, ^e^250 bar; (n = 6).

Quali-quantitative information on the individual fatty acids that compose the lipid classes of wheat germ oils were obtained by GC and HPLC analyses. Table 1 shows the fatty acids composition, expressed as percentage of total fatty acids, of oil samples obtained by using organic solvents and SC-CO_2_. In all samples the main fatty acids were palmitic (16:0), oleic (18:1 n-9), linoleic (18:2 n-6), linolenic (18:3 n-3), and eicosaenoic (20:1 n-9). Table 1 also shows results for saturated (SFA), monounsaturated (MUFA), and polyunsaturated (PUFA) fatty acids. PUFA were the main group of fatty acids in all analysed samples, ranging from 57-60%, and values of about 18% and 21-23% were observed for SFA and MUFA, respectively. A one-way ANOVA showed significant differences (p<0.05) among different extraction procedures in particular for oleic, linoleic, linolenic, arachic (20:0), and eicosaenoic fatty acids. It also showed significant differences among different extraction procedures for monounsaturated and polyunsaturated fatty acids. Methanol extract showed the lowest value for MUFA and the highest value for the PUFA fraction. The amounts, expressed as μg/mg oil, of the main unsaturated fatty acids (18:1, 18:2, and 18:3 n-3) in all wheat germ oils and are reported in Figure 4. Significant differences (p<0.05) were observed among different extraction procedures. Wheat germ oil from MeOH extraction showed the lowest amounts of unsaturated fatty acids (101.7, 230.3, and 36.4 μg/mg for 18:1 n-9, 18:2 n-6, and 18:3 n-3, respectively, as mean values over six samples), whereas oil obtained at 250 bar exihibited the highest levels, showing values of 188.4, 409.1, and 59.5 μg/mg for 18:1 n-9, 18:2 n-6, and 18:3 n-3, respectively.

**Table 1:**
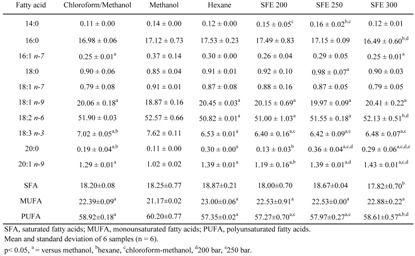
Fatty acids composition (expressed as % of total fatty acids), measured by a gas chromatography-flame ionization detection (GC-FID) method, of wheat germ oil obtained by different extraction procedures.

The oxidation status of fatty acids was also measured by HPLC detection of conjugated dienes fatty acids hydroperoxides (HP). The level of the oxidative products HP in the oils extracted by SC-CO_2_ ranged from 12 to 14 nmol/mg oil, values comparable to those measured in oils obtained by solvent extraction (10-15 nmol/mg oil), and prevalently derived from 18:2 n-6 degradation, due to the great content of this fatty acid and the high stability of its hydroperoxides [19].

Our results confirm previous reports indicating no significant quality differences in SC-CO_2_ extracted wheat germ oil compared to solvent extracted oil. In conclusion, supercritical carbon dioxide extraction has immediate advantages over traditional extraction techniques: it is a flexible process due to the possibility of continuous modulation of the solvent power/selectivity of the SC-CO_2_, allows the elimination of polluting organic solvents and of the expensive post-processing of the extracts for solvent elimination.

## 3. Experimental

### 3.1. Materials

All solvents used, of the highest available purity, were purchased from Merck (Darmstadt, Germany). Triolein, trilinolein, fatty acids and fatty acids methyl esters standards, and desferal (deferoxamine mesylate salt) were purchased from Sigma–Aldrich (Milan, Italy). The reagent 20% BF_3_ in MeOH was purchased from Supelco (Bellefonte, PA, USA). *cis,trans*-13-Hydroperoxy-octadecadienoic acid (c,t-13-HPODE) and *cis,trans*-9-hydroperoxyoctadecadienoic acid (c,t-9-HPODE) were purchased from Cascade (Cascade Biochem. Ltd., London, U.K.). All other chemicals used in this study were of analytical grade. Flaky food-grade wheat germ was supplied from Sos Ortos (Ozieri, Italy). Before using the vegetable matter was ground with a Malavasi mill (Bologna, Italy) and the particle sizes were in the 180-250 μm range.

### 3.2. Solvent Extraction

Total lipids were extracted from aliquots (100 mg) of wheat germ by addition of CHCl_3_/MeOH (12 mL, 2/1, v/v) solution [20], or MeOH (8 mL) at room temperature, in order to avoid fatty acids oxidation. After filtration, aliquots of the CHCl_3_ or MeOH phase (250 μL) were collected and total lipids were quantified by the method of Chiang, Gessert and Lowry [21]. Solvent extraction was also performed with hexane in a Soxhlet apparatus for 16 h, as standard and reference method for the extraction of fats and oils from food matrices.

### 3.3. Apparatus for SFE

Supercritical CO_2_ extractions were performed in a laboratory apparatus [22,23], equipped with a 400 cm^3^ extraction vessel and two separator vessels of 300 and 200 cm^3^ respectively connected in series. Extraction was carried out in a semi batch mode: batch charging of vegetable matter and continuous flow solvent. The extraction of the wheat germ oil was obtained working at 40°C at 200, 250 and 300 bar in the extraction vessel and by using only one separator (at 20 bar and 15°C ) to recover the extract.

### 3.4. Preparation of α-tocopherol and fatty acids

Separation of fatty acids and α-tocopherol was achieved by mild saponification [24] as follows: dried aliquots (3 mg) of the CHCl_3_, MeOH, and hexane extracts and the fixed oil (3 mg) were dissolved in EtOH (5 mL) and then Desferal solution (100 μL, 25 mg/mL of H_2_O), aqueous ascorbic acid solution (1 mL, 25% w/v), and 10 N KOH (0.5 mL) were added. The mixtures were left in the dark at room temperature for 14 h. After addition of n-hexane (10 mL) and H_2_O (7 mL), samples were centrifuged in a 4237R refrigerated centrifuge (ALC, Milan, Italy) for 1 h at 900*g*. The hexane phase with vitamin E was collected, the solvent was evaporated in a rotary evaporator (Rotavapor R-114, Büchi Labortechnik, Flawil, Switzerland) under reduced pressure, the residue was dissolved in MeOH (500 μL) and aliquots of the samples were injected into the HPLC system. After addition of further *n*-hexane (10 mL) to the mixtures, samples were acidified with 37% HCl to pH 3-4 and then centrifuged for 1 h at 900*g*. The hexane phase with free fatty acids and hydroperoxides was collected and the solvent was evaporated. A portion of the dried residue was dissolved in CH_3_CN (500 μL) with 0.14% CH_3_COOH (v/v) and aliquots of the samples were injected into the HPLC system. 

Aliquots of dried fatty acids was methylated with 14% BF_3_ in MeOH (1 mL) [25] for 30 min at room temperature. After addition of n-hexane (4 mL) and H_2_O (2 mL) samples were centrifuged for 20 min at 900*g*. The hexane phase with fatty acids methyl esters was collected, the solvent was evaporated, the residue was dissolved in n-hexane (100 μL) and aliquots of the samples were injected into the GC system. The recovery of fatty acids during saponification was calculated by using an external standard mixture. All solvents evaporation was performed under vacuum. 

### 3.5. HPLC analysis

Analysis of unsaturated fatty acids was carried out with an Agilent Technologies 1100 liquid chromatograph. Analyses of unsaturated fatty acids and conjugated diene fatty acid hydroperoxides (HP)[24], detected at 200 and 234 nm respectively, were carried out with a Chrompack column, Inertsil 5 ODS-2 (150 mm × 4.6 mm, 5 μm particle size) with a mobile phase of CH_3_CN/H_2_O/CH_3_COOH (70/30/0.12, v/v/v) at a flow rate of 1.5 mL/min. The identification of the peaks was made using standard compounds and second derivative as well as conventional UV spectra, generated using the Agilent Chemstation A.10.02 software. The amount of α-tocopherol was measured by electrochemical detection [24], using a Thermo Separation Products (Milan, Italy) P1000 pump equipped with an electrochemical detector INTRO (Antec Leyden, Leiden, The Netherlands). An automatic injector, Triathlon (Spark Holland BV, AJ Emmen, The Netherlands) was used. A C-18 Hewlett Packard ODS Hypersil column, 5 μm particle size, 100 × 2.1 mm, was used with a mobile phase of MeOH/CH_3_COONa 0.05M pH 5.5 (95/5, v/v) at a flow rate of 0.3 mL/min. The electrochemical detector was set at an oxidizing potential of 0.6 V. Data were collected and analysed using the Agilent Chemstation A.10.02. software. Quantification of vitamin E was performed using a standard reference curve.

### 3.6. GC analysis

Fatty acid methyl esters were measured on a Hewlett-Packard HP-6890 gas chromatograph (Hewlett-Packard, Palo Alto, CA, USA) equipped with a flame ionisation detector and a cyanopropyl methyl-polysiloxane HP-23 FAME column (30 m x 0.32 mm x 0.25 μm) (Hewlett-Packard) [26]. Nitrogen was used as carrier gas at a flow rate of 2 mL/min. The oven temperature was programmed from 45 °C to 175 °C at a rate of 80 °C/min and held for 45 min; injector temperature was set at 250 °C, and detector temperature at 300 °C. The fatty acid methyl esters were identified by comparing the retention times with those of standard compounds. The percentage composition of individual fatty acids were calculated using a calibration curve with components injected at different concentrations, using the Hewlett-Packard A.05.02 software. 

### 3.7. Statistical analyses

Graph Pad INSTAT software (GraphPad software, San Diego, CA, USA) was used to calculate the means and standard deviations of three independent experiments involving duplicate analyses for each sample/condition (n=6). Means of results were compared by one-way analysis of variance (One-way ANOVA) using the Tukey-Kramer Multiple Comparisons Test. Probability values of p < 0.05 were considered as significant.
